# Retrospective Comparison of Short- and Mid-Term Performance of 200-µm Polyethylene Glycol Microspheres vs Ethiodized Oil Emulsion for Genicular Artery Embolization in Symptomatic Knee Osteoarthritis

**DOI:** 10.1007/s00270-025-04290-6

**Published:** 2025-12-14

**Authors:** Wali Badar, Layth Alkhani, Faisal Al-Qawasmi, Qian Yu, Magdalena Anitescu, Brendon Ross, Sara Wallace, Mikin Patel, Osman Ahmed

**Affiliations:** 1 Department of Radiology, University of Illinois at Chicago, Chicago, IL USA; 2 Department of Engineering, Stanford University, Palo Alto, CA USA; 3 College of Medicine, University of Illinois at Peoria, Peoria, IL USA; 4 Department of Radiology, University of Chicago, Chicago, IL USA; 5 Department of Anesthesia, University of Chicago, Chicago, IL USA; 6 Department of Orthopedic Surgery and Rehabilitation Medicine, University of Chicago, Chicago, IL USA; 7 Joint and Vascular Institute, Libertyville, IL USA; 8 Department of Radiology, University of Utah, Salt Lake City, IL USA

**Keywords:** Genicular artery embolization, Microspheres, Lipiojoint, WOMAC, Knee osteoarthritis

## Abstract

**Purpose:**

To report up to 6-month outcomes of genicular artery embolization (GAE) using polyethylene glycol microspheres vs ethiodized oil emulsion in patients with medically refractory symptomatic knee osteoarthritis (KOA).

**Material and Methods:**

In this single-center retrospective study, sixty-eight patients (65 y.o, M/F (11/57)) underwent GAE from 9/2021 to 3/2025 using a permanent agent: 200-micron microspheres (HydroPearl®, n = 42), or a temporary agent: emulsion of ethiodized oil (LipioJoint, n = 46). Outcomes were assessed with the Western Ontario and McMaster Universities Osteoarthritis Index (WOMAC) total and Pain sub-score at baseline, 1, 3, and 6 months post-GAE. Adverse events (AEs) were reported by the CIRSE AE classification system. A descriptive statistical and multivariable analysis was performed.

**Results:**

Eighty-eight knees treated with KOA severity: Kellgren–Lawrence (KL) 2: 10 vs 23%, KL 3: 57 vs 33%, KL4: 33 vs 44%, respectively (permanent vs temporary, *P* = 0.051). Pain sub-scale score decreased by 27 vs 44%, 25 vs 37%, and 23 vs 36% at 1, 3, and 6 months, respectively. There was higher percent WOMAC pain reduction in ethiodized oil emulsion at 1 but not at 3 and 6 months (*P* = 0.020–0.119). Adverse events included: skin changes without ulceration (n = 14), knee swelling requiring prednisone (n = 5), and access site hematoma (n = 2). A lower AE rate was observed for total AEs and skin changes with ethiodized oil emulsion (*P* = 0.018).

**Conclusion:**

GAE shows positive short-term outcomes up to 6 months post-treatment for KOA with temporary and permanent embolic agents. Ethiodized oil may offer a better short-term efficacy and lower risk of skin discoloration compared to polyethylene glycol microspheres.

## Introduction

Knee osteoarthritis (KOA) affects more than 595 million patients worldwide, with predictions estimating that its prevalence will increase by 74.9% from 2020 by 2050 [1]. Estimates suggest that over half of patients experience inadequate relief from conservative treatments, with a portion of patients further excluded from surgical candidacy [2]. KOA was originally believed to be the natural progression of degenerative wear and tear; however, evidence has recently suggested a more complex pathology, with synovitis playing a strong role [3, 4]. Genicular artery embolization (GAE) is an established procedure founded on this new understanding of KOA to provide an alternative avenue for relief in this specific group of patients, as GAE involves selection and embolization of genicular arteries to reduce synovial arterial hypervascularity.

Embolic selection is pivotal in various embolization procedures and presumably as important for successful GAE outcomes. Permanent microspheres have been investigated in GAE literature, boasting impressive clinical outcomes [5]. However, the permanent nature of the embolic stirs natural concern for potential off-target embolization and potential downstream adverse outcomes [6]. Ethiodized oil is a temporary embolic agent that was originally approved by the FDA for use in treating hepatocellular carcinoma with transarterial chemoembolization [7]. More recently, Sapoval et al. has described it as an efficacious, transient embolic for GAE [8]. Its non-uniform, soft, and compressible characteristics allow the oil to redistribute and eventually allow embolized arterioles to recanalize over days to weeks. Its evanescent nature is hypothesized to provide a better safety profile in comparison with permanent embolic agents [8]. Two meta-analyses by Chlorogiannis et al. and Epelboym et al. have shown no significant differences in efficacy and safety outcomes between permanent and temporary embolic agents [9, 10]. Despite this high level of evidence, heterogeneity exists between existing studies with respect to selection of permanent and temporary embolic material, as most reported outcomes with temporary embolic use imipenem/cilastatin. Therefore, further study is imperative to understand the comparative performance of embolic type.

In this study, we aim to explore the efficacy and safety of permanent microspheres vs ethiodized oil in GAE at our institution.

## Methods

### Study Design

**Fig. 1:**
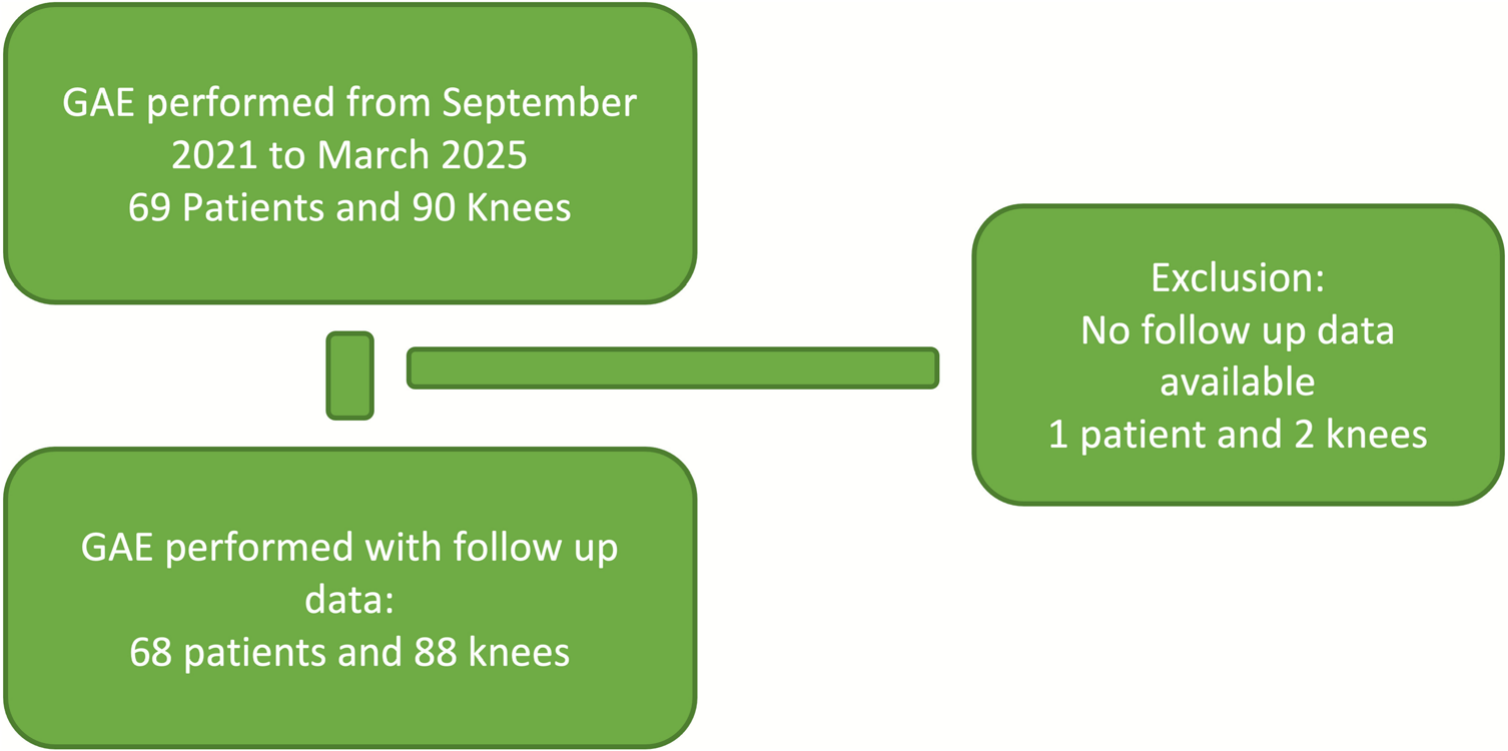
Flow chart illustrates the inclusion and exclusion criteria of patients in this study

In this single-center retrospective study, 69 consecutive patients who received GAE for symptomatic KOA between September 2021 and March 2025 were included in this single-center, IRB-approved retrospective study (#BLINDED). The study period included was based on the first GAE performed and the final date in which sufficient follow-up clinical data (i.e., six-month follow-up) was available for the study. Patients with osteoarthritis refractory to conservative medical therapy for greater than six months (i.e., activity modification, NSAIDs, acetaminophen, oral opiate pain medicines, intra-articular injections, and/or oral/injectable corticosteroids) were offered GAE and included in this cohort. Exclusion criteria included subjects with rheumatoid or seronegative arthritis, peripheral arterial disease, chronic kidney disease with a glomerular filtration rate < 30 mL/min/1.73 m^2^, uncorrectable coagulopathy, iodinated contrast allergy, or septic arthritis. Three patients received GAE due to refractory pain after knee arthroplasty. These patients received treatment with temporary embolic material and were included in the study analysis. Baseline data collected for patients included Kellgren–Lawrence (KL) grading of knee radiographs and Western Ontario and McMaster Universities Osteoarthritis Index (WOMAC). One patient treated with temporary embolic material was excluded from data analysis because of insufficient follow-up data. Inclusion and exclusion criteria are included in Fig. 1. Outcomes of 44 patients in this study have been previously reported [11–13].

### Embolization technique

**Fig. 2:**
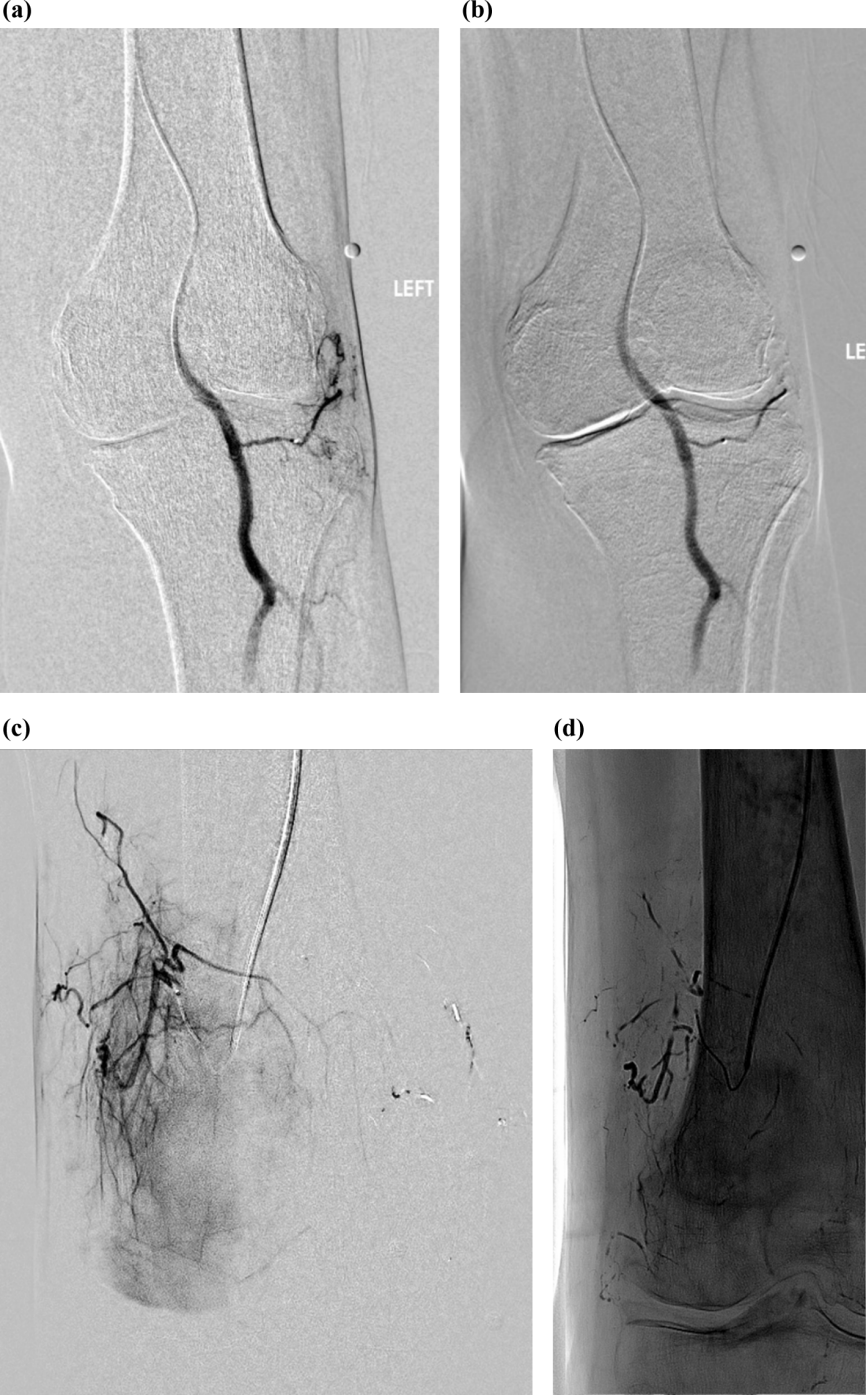
**a** Hyperemia is noted from the left inferior lateral genicular artery after selective catheterization. **b** Resolution of hyperemia in the left inferior lateral genicular artery after embolization with permanent polyethylene glycol microspheres. **c** Hyperemia is noted from the right superior lateral genicular artery after selective catheterization. **d** Resolution of hyperemia in the right superior lateral genicular artery after embolization with temporary ethiodized oil emulsion

Each GAE procedure was performed by a single fellowship trained, board-certified, interventional radiologist (BLINDED) with 15 years of experience. The embolization technique, previously described in other literature, was performed using 200-μm microspheres (HydroPearl®, Tokyo Japan) or an emulsion of ethiodized oil (Lipiodol®) and ioversol contrast [9]. For microsphere embolization, the 200-μm microspheres were mixed in a 1:2 dilution with iodinated contrast for a total volume of 15 ml. For the ethiodized oil emulsion, the ethiodol was mixed with ioversol (Optiray 320, Guerbet) in a 3:1 ratio. The number of arteries and volume of embolic agent were recorded for each treatment. Embolization was performed using the pruning technique where hyperemia arising off of the parent genicular arteries was treated while maintaining flow in the parent artery as described previously [11]. Examples of pre- and post-embolization digital subtraction images are seen for temporary and permanent embolic agents in Fig. 2a-d. There was an institutional transition from permanent embolic material to temporary embolic material in March of 2024 due to reported safety and efficacy of these agents [8].

Patients were observed in the short-stay unit for four hours post-procedure before being discharged home. Patients were continually assessed for adverse events (AEs) at 1 day post-procedure via telehealth calls and during the first month post-procedure. AE reporting was done following the published Cardiovascular and Interventional Radiological Society of Europe (CIRSE) classification system [14]. Clinic follow-up visits took place at 1 week, 1 month, 3 months, and 6 months post-procedure. The primary endpoint for this study was a reduction of KOA pain as measured by WOMAC total (0–96) and WOMAC pain scoring (0–20) at 1, 3, and 6 months post-GAE as compared to baseline.

### Outcomes

Technical success was defined by catheterization of at least one abnormal genicular artery with the improvement of hyperemic blush with embolization. Primary clinical outcome recorded was WOMAC total score and pain score reduction at one and three months. Adverse events were recorded up to one month after treatment [15].

### Statistical Analysis

Descriptive statistics were used to present demographic and procedural data. Continuous data were represented with mean and standard deviation, and categorical data were presented with a proportion. Descriptive statistical analysis was performed to investigate differences between temporary and permanent embolic cohorts with Fisher and Student t-tests. Multivariable analysis was performed for descriptive predictors with *P*- value < 0.100 using binomial regression. All statistical analysis was performed using MATLAB (MathWorks, Natick MA). P-value of 0.05 was selected for significance.

## Results

A total of 68 patients met the inclusion/exclusion criteria with an age of 65.2 ± 15.1 and were predominantly female (84%, 57/68). The treatment population was predominantly African-American (57%, 39/68) with a BMI of 35.1 ± 9.8. A total of 88 knees were treated (R: 46 and L: 42). Radiographic severity distribution of knee severity was as follows: KL Grade 2 (N = 14, 16%), KL Grade 3 (N = 38, 43%), and KL Grade 4 (N = 33, 38%). These findings are summarized in Table 1. There were no differences in age (65.7 ± 13.0 vs 64.8 ± 17.0, *P* = 0.766), gender (79% vs 86% female, *P* = 0.532), race (58% vs 56% African-American, P = 0.981), or BMI (35.4 ± 9.6 vs 34.9 ± 10.2, *P* = 0.814). There was a similar degree of KL Grade 2 (23% vs 10%, *P* = 0.051) in subjects treated with ethiodized oil compared to microspheres with descriptive analysis; however, there was a significant difference in multivariate regression analysis (*P* = 0.029). Results of univariate and multivariate analysis are seen in Table 2.

**Table 1:** Baseline characteristics of 68 patients

Age (y.o.), mean ± SD		65.2 ± 15.1
Gender, n (%)	Male	11 (16)
	Female	57 (84)
Race, n (%)	African-American	39 (57)
	White	27 (40)
	Asian	2 (3)
BMI, mean ± SD		35.1 ± 9.8
Total number of knees treated, n (%)	Right	46 (52)
	Left	42 (48)
KL grade, n (%)	Grade 2	14 (16)
	Grade 3	38 (43)
	Grade 4	33 (38)
Number of arteries embolized per procedure, mean ± SD		3.3 (0.8)
Type of embolic used, n (%)	Permanent (microspheres)	42 (48)
	Temporary (ethiodized oil + ioversol)	46 (52)

Baseline and Post-Treatment WOMAC		
Characteristic	Mean ± SD	p-value
Baseline total WOMAC	61.9 ± 18.0	
1 Month % reduction	30.4 ± 38.3	P < 0.001*
3 Month % reduction	21.6 ± 46.3	P < 0.001*
6 Month % reduction	23.6 ± 32.4	P < 0.001*
Baseline WOMAC Pain	12.6 ± 3.5	
1 Month % reduction	35.5 ± 32.8	P < 0.001*
3 Month % reduction	30.4 ± 32.0	P < 0.001*
6 Month % reduction	28.2 ± 31.7	P < 0.001*

**Table 2:** Baseline characteristics of 68 patients stratified by embolic agents

Variable		Permanent	Temporary	p-value univariate	p-value multivariate
Age (y.o.), mean ± SD		65.7 ± 13.0	64.8 ± 17.0	0.766	
Gender, n (%)	*Male*	7 (21)	5 (14)	0.532	
	*Female*	27 (79)	32 (86)		
Race, n (%)	*African-American*	19 (58)	20 (56)	0.981	
	*White*	13 (39)	15 (42)		
	*Asian*	1 (3)	1 (3)		
BMI, mean ± SD		35.4 ± 9.6	34.9 ± 10.2	0.814	
Total number of knees treated, n (%)	*Right*	21 (50)	25 (54)	0.827	
	*Left*	21 (50)	21 (46)		
KL grade, n (%)	*Grade 2*	4 (10)	10 (23)	0.051	0.029**
	*Grade 3*	24 (57)	14 (33)		
	*Grade 4*	14 (33)	19 (44)		
Number of arteries embolized per procedure, mean ± SD		3.1 ± 0.8	3.5 ± 0.8	0.028**	0.028**

Baseline and post-treatment WOMAC stratified by embolic			
Characteristic	Mean ± SD		p-value
	Permanent	Temporary	
Baseline WOMAC pain	12.6 ± 3.2	12.5 ± 3.9	0.923
% reduction from baseline at one month	27.2 ± 31.6	43.6 ± 32.3	0.020**
% reduction from baseline at three months	24.9 ± 32.2	37.3 ± 30.9	0.097
% reduction from baseline at six months	23.1 ± 29.9	35.5 ± 33.3	0.119

GAE: Genicular artery embolization; KL: Kellgren–Lawrence; BMI: body mass index; WOMAC: Western Ontario and McMaster Universities Osteoarthritis Index

A total of 288 vessels were treated with 3.2 ± 0.8 vessels per GAE. Of the 88 knees treated, 48% (42/88) were treated with microspheres and 52% (46/88) were treated with ethiodized oil emulsion. An average volume of 1.9 ± 0.7 ml of embolic material was used per GAE. More vessels were treated during ethiodized oil emulsion embolization compared to microsphere treatment (3.5 ± 0.8 vs 3.1 ± 0.8, *P* = 0.015). This was confirmed with multivariable analysis (*P* = 0.028). No differences were observed in embolic volume of treatment per GAE between the two embolics (1.9 ± 0.8 vs 1.8 ± 0.5, *P* = 0.352). The most common vessel treated was the lateral inferior genicular artery (71/288, 25%) followed by the medial inferior genicular artery (64/288, 22%), lateral superior genicular artery (59/288, 20%), descending genicular artery (52/288, 18%), medial superior genicular artery (32/288, 11%), and lastly the anterior tibial recurrent artery (10/288, 4%).

**Fig. 3:**
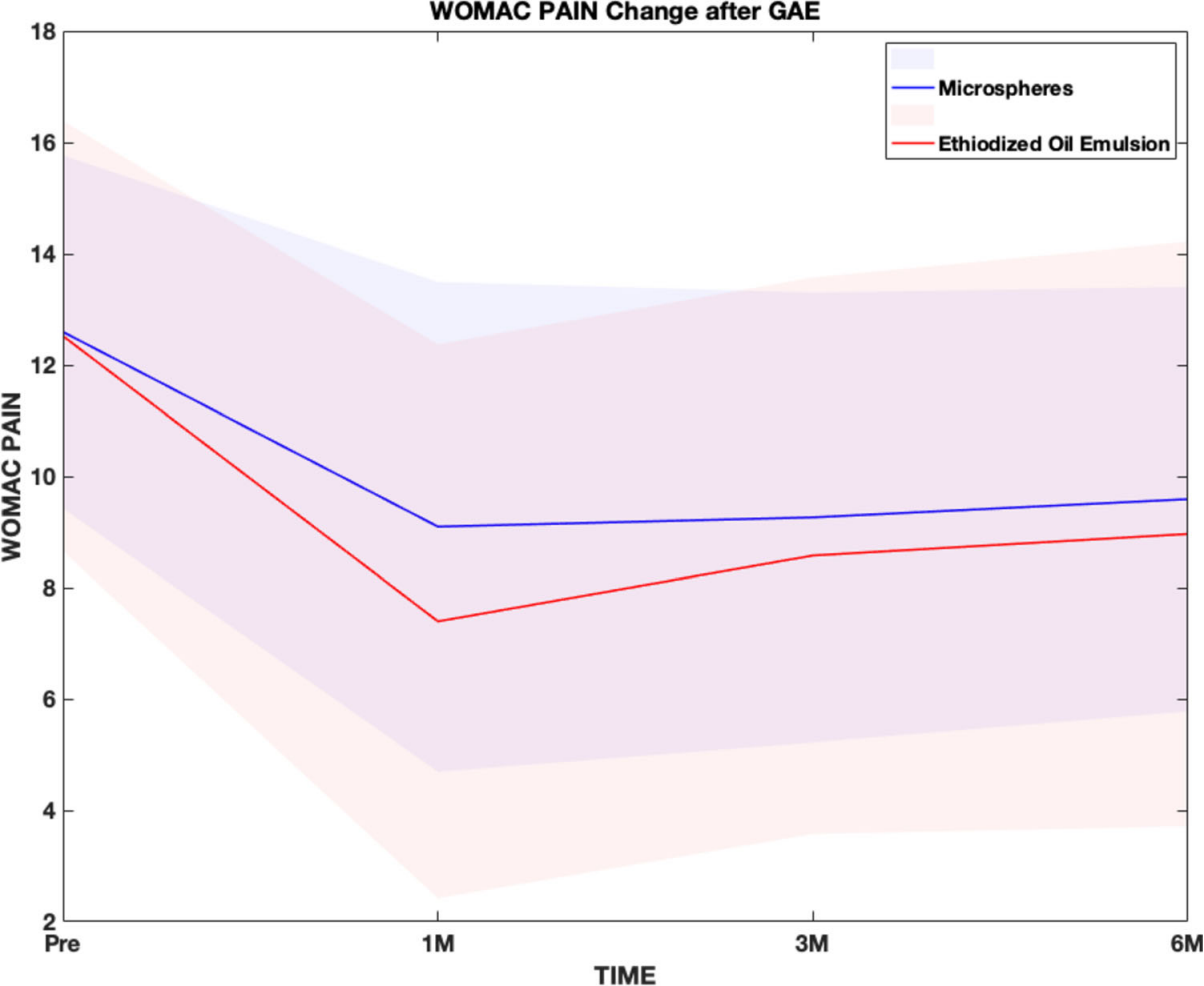
Comparative changes in WOMAC pain score overtime with permanent and temporary embolic agents. Significant difference in WOMAC pain reduction is seen at 1 month with greater pain reduction in lipiodol emulsion compared to microspheres (P = 0.020)

The average baseline total WOMAC score was 61.9 ± 18.0 with a 30.4% reduction at one month, a 21.6% reduction at three months, and a 23.6% reduction at six months (*P* < 0.001). The average baseline WOMAC pain score was 12.6 ± 3.5 with a 35.5% reduction at one month, a 30.4% reduction at three months, and a 28.2% reduction at six months (*P* < 0.001). No difference was identified between baseline WOMAC pain (12.6 ± 3.2 vs 12.5 ± 3.9) scores between the two embolic groups (*P* = 0.923). A significant difference was identified in WOMAC pain score reduction at one month (27.2 ± 31.6 vs 43.6 ± 32.3) but not at three months (24.9 ± 32.2 vs 37.3 ± 30.9) or six months (23.1 ± 29.9 vs 35.5 ± 33.3) between the two embolic groups (*P* = 0.020–0.119). These findings are summarized in Table 2 and Fig. 3.

The adverse event rate was 22% (19 of 88 knees). The most common adverse event was skin discoloration without ulceration (16%, 14 of 88 knees) followed by knee swelling post-GAE requiring prednisone (6%, 5 of 88) and then access site hematoma (2%, 2 of 88). There was a lower total adverse event rate in ethiodized oil emulsion embolization (11%, 5 of 46) compared to microsphere treatment (33%, 14 of 42) (*P* = 0.018). A lower adverse skin discoloration rate was observed in ethiodized oil emulsion embolization (7%, 3 of 46) compared to microsphere treatment (26%, 11 of 42) (*P* = 0.018). No differences were observed in knee swelling and access site hematoma rates (*P* = 0.188–0.999). Most adverse events were self-resolving with conservative therapy and were characterized as Grade 1 by the CIRSE adverse event classification system; however, instances of knee swelling requiring prednisone were Grade 2 [14]. These findings are summarized in Table 3.

**Table 3:** Adverse events up to one month post-GAE (N = 88)

	n	%	
Skin changes	14	16%	
Knee swelling requiring prednisone	5	6%	
Access site hematoma	2	2%	
Total adverse events	19	22%	
Subset analysis of adverse events by embolic selection			
	Permanent (n, %)	Temporary (n, %)	P-Value
Skin changes	11 (26%)	3 (7%)	0.018
Knee swelling requiring prednisone	4 (10%)	1 (2%)	0.188
Access site hematoma	1 (2%)	1 (2%)	0.999
Total adverse events	14 (31.0%)	5 (11.9%)	0.018

GAE: Genicular artery embolization

## Discussion

The current study established single-center, short- and middle-term outcomes using both permanent and temporary embolic material. This study demonstrated durable short- and mid-term outcomes of GAE for symptomatic KOA using both temporary and permanent embolic materials with an acceptable adverse event profile. The percent reduction in WOMAC score generally decreased with time, possibly indicating the limited durability of the treatment, but the observed trend generally agreed with published outcomes [16, 17]. The current study differed from the reported outcomes of ethiodized oil emulsion by Guzelbey et al. and Sapoval et al. with a weaker response at 1 and 3 months [8, 18]. The current study had a higher average BMI than Sapoval et al. (31.0 vs 34.9) and greater radiographic severity than Guzelbey et al. (38% of KL ≥ 3 vs 77% of KL ≥ 3), which may explain the difference in outcomes. Furthermore, the current study highlighted the improved short-term efficacy at 1 month of ethiodized oil emulsion compared to permanent microspheres with reduced risk of skin discoloration. On average, more vessels were treated per GAE with the ethiodized oil emulsion compared to permanent microspheres, which may explain the improved short-term efficacy, but this did not come at the expense of increased skin discoloration. Additionally, there was a higher degree of radiographic severity in subjects treated with microspheres, which may explain the worse short-term outcomes, as the difference in degree of radiographic severity between the two embolic cohorts achieved statistical significance on multivariable analysis. Further investigation is necessary with long-term outcomes as well as larger sample sizes. Additionally, randomized control trials are necessary to understand the true advantage of one agent over the other.

Few studies have investigated the comparative effects of temporary vs permanent embolic agents for GAE. In 2018, Bhatia et al. evaluated the short- and long-term outcomes of GAE for KOA using 100–300 trisacryl gelatin microspheres vs imipenem/cilastatin in 14 patients and observed no difference in clinical response defined by a reduction in WOMAC pain score by 50% at three and 24 months [19]. The authors did not report safety outcomes. The current study was larger in sample size and reported the different outcomes for short-term clinical response based on embolic agent. This can be explained by either a difference in the selection of embolic material, polyethylene glycol and ethiodized oil emulsion vs 100–300 trisacryl gelatin microspheres and imipenem/cilastatin, or different treatment populations/technical factors. Bhatia et al. had a similar distribution of radiographic severity of KOA between the treatment groups, while the current study had lower radiographic severity of KOA in the temporary embolic cohort. The Trial Genicular Artery Embolization Using Imipenem/Cilastatin vs. Microsphere for Knee Osteoarthritis: Randomized Clinical Trial (GAUCHO) offers high level evidence for the comparative performance of temporary (imipenem/cilastatin) and permanent agents (trisacryl gelatin) [20, 21]. The study has recently published a preliminary scientific abstract, including three- and 12-month outcomes, and has noticed no difference in clinical outcomes (50% reduction in WOMAC pain and 10-point reduction in KOOS) or skin discoloration after treatment in 82 knees. Future publication of complete results will be imperative for understanding comparative outcomes. The current study differs in study design and investigates different agents for treatment. Although the current study identified a comparative benefit of ethiodized oil emulsion compared to microspheres at 1 month, this time interval was not evaluated in the GAUCHO trial. Furthermore, the current results agreed with the 3-month outcomes of the GAUCHO trial. To date, two authors, Chlorogiannis et al. and Epelboym et al., have performed a systematic review to evaluate the comparative benefit of temporary vs permanent embolic materials up to 12 months after GAE [9, 10]. These authors identified no differences in comparative benefit; however, the majority of studies used imipenem/cilastatin for temporary agents. There was a high degree of heterogeneity in the subset analyses, indicating variability in the reported data. Chlorogiannis et al. reported no differences in transient skin color changes between the two embolic groups. The current study differed in one-month outcomes between embolic types, but this can be explained by differences in patient selection (i.e., radiographic severity) as well as treatment characteristics (i.e., vessels treated). Currently, it is believed that imipenem/cilastatin and ethiodized oil emulsion work with a similar method of temporary occlusion, so it is unclear why one agent would be more effective than the other, and thus, further investigation is necessary [22].

This study had limitations inherent to retrospective, single-center design. The outcomes of this study are only generalizable to the specific embolic agents that were used (ethiodized oil emulsion and 200-µm polyethylene glycol microspheres) and their treatment characteristics (dilution, volume, etc.). Furthermore, the embolic agent cohorts were different in terms of radiographic severity, which may confound outcomes. The two study populations were not matched and therefore highly susceptible to selection and allocation bias. Ethiodized oil emulsion was used in later years, while microspheres were used in earlier years, and therefore, outcomes may be influenced by an operator experience/learning curve. The current study only evaluated short- and middle-term outcomes, and therefore, conclusions cannot be made about the durability of different embolic agents like those made in the GAUCHO trial. Future randomized control trials are necessary with long-term follow-up to understand efficacy and durability of temporary and permanent embolic agents.

GAE using ethiodized oil emulsion and 200-µm polyethylene glycol microspheres are both safe and effective in the short term. Temporary embolic agents may offer an improved clinical response at one month post-GAE and improve safety profile in comparison with permanent agents with respect to skin discoloration. Future randomized control trials are needed to assess the utility of different permanent and temporary embolic agents.

## References

[1] GBD 2021 Osteoarthritis Collaborators. 2023 Global, regional, and national burden of osteoarthritis, 1990–2020 and projections to 2050: a systematic analysis for the Global Burden of Disease Study 2021 Lancet Rheumatol. 5 9 e508 e522 10.1016/S2665-9913(23)00163-7 10.1016/S2665-9913(23)00163-7 PMC10477960 37675071

[2] Conaghan PG, Peloso PM, Everett SV, Rajagopalan S, Black CM, Mavros P, et al. Inadequate pain relief and large functional loss among patients with knee osteoarthritis: evidence from a prospective multinational longitudinal study of osteoarthritis real-world therapies. Rheumatology (Oxford). 2015;54(2):270–7. 10.1093/rheumatology/keu332. 25150513 10.1093/rheumatology/keu332 PMC4301711

[3] Berenbaum F. Osteoarthritis as an inflammatory disease (osteoarthritis is not osteoarthrosis!). Osteoarthritis Cartilage. 2013;21(1):16–21. 10.1016/j.joca.2012.11.012. 23194896 10.1016/j.joca.2012.11.012

[4] Taslakian B, Miller LE, Mabud TS, Macaulay W, Samuels J, Attur M, et al. Genicular artery embolization for treatment of knee osteoarthritis pain: Systematic review and meta-analysis. Osteoarthr Cartil Open. 2023;5(2):100342. 10.1016/j.ocarto.2023.100342. 36865988 10.1016/j.ocarto.2023.100342 PMC9971280

[5] Padia SA, Genshaft S, Blumstein G, Plotnik A, Kim GHJ, Gilbert SJ, et al. Genicular artery embolization for the treatment of symptomatic knee osteoarthritis. JB JS Open Access. 2021;6(4):2100085. 10.2106/JBJS.OA.21.00085. 10.2106/JBJS.OA.21.00085 PMC8542160 34703964

[6] Lee S, Ghosh A, Xiao N, Gordon AC, Heidarpour N, Funaki B, et al. Embolic agents: particles. Semin Intervent Radiol. 2023;40(3):315–22. 10.1055/s-0043-1769744. 37565087 10.1055/s-0043-1769744 PMC10410675

[7] Idée JM, Guiu B. Use of Lipiodol as a drug-delivery system for transcatheter arterial chemoembolization of hepatocellular carcinoma: a review. Crit Rev Oncol Hematol. 2013;88(3):530–49. 10.1016/j.critrevonc.2013.07.003. 23921081 10.1016/j.critrevonc.2013.07.003

[8] Sapoval M, Querub C, Pereira H, Pellerin O, Boeken T, Di Gaeta A, et al. Genicular artery embolization for knee osteoarthritis: results of the LipioJoint-1 trial. Diagn Interv Imaging. 2024;105(4):144–50. 10.1016/j.diii.2023.12.003. 38102013 10.1016/j.diii.2023.12.003

[9] Chlorogiannis DD, Vasilopoulou A, Konstantinidis CI, Pagona AE, Filippiadis DK. Knee pain improvement after genicular artery embolization for the management of knee osteoarthritis: an updated systematic review and meta-analysis of 21 studies. Radiologie (Heidelb). 2024;64(Suppl 1):32–46. 10.1007/s00117-024-01388-9. 39527285 10.1007/s00117-024-01388-9

[10] Epelboym Y, Mandell JC, Collins JE, Burch E, Shiang T, Killoran T, et al. Genicular artery embolization as a treatment for osteoarthritis related knee pain: a systematic review and meta-analysis. Cardiovasc Intervent Radiol. 2023;46(6):760–9. 10.1007/s00270-023-03422-0. 36991094 10.1007/s00270-023-03422-0

[11] Badar W, Anitescu M, Ross B, Wallace S, Uy-Palmer R, Ahmed O. Quantifying change in perfusion after genicular artery embolization with parametric analysis of intraprocedural digital subtraction angiograms. J Vasc Interv Radiol. 2023;34(12):2190–6. 10.1016/j.jvir.2023.08. 37673399 10.1016/j.jvir.2023.08.041

[12] Badar W, Al-Qawasmi F, Alkhani L, Varadhan A, Sajan A, Yu Q, et al. One- and two-year outcomes of genicular artery embolization for symptomatic knee osteoarthritis: a single-center, retrospective study using 200-µm polyethylene glycol microspheres. Cardiovasc Intervent Radiol. 2025;48:1–9. 10.1007/s00270-025-04163-y. 40847222 10.1007/s00270-025-04163-y

[13] Badar W, Alkhani L, Al-Qawasmi F, Varadhan A, Husnain A, Khan M, et al. Prognostication of three-month genicular artery embolization outcomes using pre-procedural MRIs. Cardiovasc Intervent Radiol. 2025;48:1–10. 10.1007/s00270-025-04159-8. 40847221 10.1007/s00270-025-04159-8 PMC12665625

[14] Filippiadis D, Pereira PL, Hausegger KA, Ryan AG, Binkert CA. CIRSE Standards of Practice for the Classification of Complications: The Modified CIRSE Classification System. Cardiovasc Intervent Radiol. 2025 Oct 16. 10.1007/s00270-025-04200-w. Epub ahead of print. PMID: 41102465. 10.1007/s00270-025-04200-w PMC12748290 41102465

[15] Ahmed O, Epelboym Y, Haskal ZJ, Okuno Y, Taslakian B, Sapoval M, et al. Society of interventional radiology research reporting standards for genicular artery embolization. J Vasc Interv Radiol. 2024;35(8):1097–103. 10.1016/j.jvir.2024.04.018. 38685470 10.1016/j.jvir.2024.04.018

[16] Cusumano LR, Sparks HD, Masterson KE, Genshaft SJ, Plotnik AN, Padia SA. Genicular artery embolization for treatment of symptomatic knee osteoarthritis: 2-year outcomes from a prospective IDE trial. J Vasc Interv Radiol. 2024;35(12):1768–75. 10.1016/j.jvir.2024.08.028. 39322180 10.1016/j.jvir.2024.08.028

[17] Okuno Y, Korchi AM, Shinjo T, Kato S, Kaneko T. Midterm clinical outcomes and MR imaging changes after transcatheter arterial embolization as a treatment for mild to moderate radiographic knee osteoarthritis resistant to conservative treatment. J Vasc Interv Radiol. 2017;28(7):995–1002. 10.1016/j.jvir.2017.02.033. 28365171 10.1016/j.jvir.2017.02.033

[18] Guzelbey T, Dablan A, Erdim C, Deniz R, Mutlu IN, Kilickesmez O. Lipiodol versus imipenem/cilastatin in genicular artery embolization: a retrospective study on safety and clinical success. Cardiovasc Intervent Radiol. 2024;47(12):1765–73. 10.1007/s00270-024-03836-4. 39160360 10.1007/s00270-024-03836-4

[19] Bhatia S, Jalaeian H, Kumar J, Acharya V, Shibuya M, Bhatia A, et al. Two-year outcomes comparing Embosphere® microspheres versus imipenem cilastatin for genicular artery embolization in patients with moderate to severe knee osteoarthritis. Knee. 2023;41:38–47. 10.1016/j.knee.2022.12.008. 36608360 10.1016/j.knee.2022.12.008

[20] Correa MP, Motta-Leal-Filho JM, Lugokeski R, Mezzomo M, Leite LR. GAUCHO - trial genicular artery embolization using imipenem/cilastatin vs. microsphere for knee osteoarthritis: a randomized controlled trial. Cardiovasc Intervent Radiol. 2022;45(7):903–10. 10.1007/s00270-022-03089-z. 35304614 10.1007/s00270-022-03089-z

[21] Correa P. Twelve months results of the Gaucho Trial: Genicular Artery embolization Using imipenem/Cilastatin vs microspHeres for chronic knee pain: a randomized controlled trial. CardioVasc Interven Radiol. 2022;45(7):903–10. 10.1007/s00270-022-03089-z 35304614

[22] Pellerin O. Are musculoskeletal embolizations a rebirth? Cardiovasc Intervent Radiol. 2024;47(12):1774–5. 10.1007/s00270-024-03860-4. 39562340 10.1007/s00270-024-03860-4

