# Correction to: Retrospective Comparison of Short and Mid Term Performance of 200 µm Polyethylene Glycol Microspheres vs Ethiodized Oil Emulsion for Genicular Artery Embolization in Symptomatic Knee Osteoarthritis

**DOI:** 10.1007/s00270-026-04476-6

**Published:** 2026-05-12

**Authors:** Wali Badar, Layth Alkhani, Faisal Al-Qawasmi, Qian Yu, Magdalena Anitescu, Brendon Ross, Sara Wallace, Mikin Patel, Osman Ahmed

**Affiliations:** 1 Department of Radiology, University of Illinois at Chicago, Chicago, IL USA; 2 Department of Engineering, Stanford University, Palo Alto, CA USA; 3 College of Medicine, University of Illinois at Peoria, Peoria, IL USA; 4 Department of Radiology, University of Chicago, Chicago, IL USA; 5 Department of Anesthesia, University of Chicago, Chicago, IL USA; 6 Department of Orthopedic Surgery and Rehabilitation Medicine, University of Chicago, Chicago, IL USA; 7 Joint and Vascular Institute, Libertyville, IL USA; 8 Department of Radiology, University of Utah, Salt Lake City, IL USA

**Correction to: Cardiovasc Intervent Radiol** 10.1007/s00270-025-04290-6

In the published version, the Ethics Declaration states the following:

Conflicts of interest

See attached ICMJE disclosure forms.

However, as no ICMJE disclosure form is provided, an erratum is to be issued with the following:

“Compliance with Ethical Standards”.

1. Funding:

“This study was not supported by any funding.”

2. Conflict of Interest:

Dr. Mikin Patel:

Leadership and Fiduciary role for Boston Scientific and Argon.

Consulting fee for Boston Scientific and Argon.

Dr. Osman Ahmed:

Grant Funding: NIH.

Consulting Fees: Medtronic, Cook, Bard, Penumbra.

Payment and Honoraria: AngioDynamics, Asahi.

Leadership and Fiduciary Role: Boston Scientific, Argon, Johnson and Johnson, Flow Medical.

Dr. Magdalena Anitescu:

Grants: Abbott, Boston Scientific, Medtronic.

Meeting Attendance Support: North American Neuromodulation Society.

3. Ethical approval:

- **For studies involving patients**, please add the following sentence:

“All procedures performed in studies involving human participants were in accordance with the ethical standards of the institutional and/or national research committee and with the 1964 Helsinki declaration and its later amendments or comparable ethical standards.”

“For this type of study formal consent is not required.”

“This study has obtained IRB approval from (University of Chicago IRB) and the need for informed consent was waived.”

**4. Informed consent**: “Informed consent was obtained from all individual participants included in the study.”

“For this type of study informed consent is not required.”

“This study has obtained IRB approval from (University of Chicago IRB) and the need for informed consent was waived.”

5. Consent for publication:

“For this type of study consent for publication is not required.”

